# Tuning of Emission Wavelength of CaS:Eu by Addition of Oxygen Using Atomic Layer Deposition

**DOI:** 10.3390/ma14205966

**Published:** 2021-10-11

**Authors:** José Rosa, Jouko Lahtinen, Jaakko Julin, Zhipei Sun, Harri Lipsanen

**Affiliations:** 1 Department of Electronics and Nanoengineering, Aalto University, P.O. Box 13500, FI-00076 Aalto, Finland; zhipei.sun@aalto.fi; 2 Department of Applied Physics, Aalto University, P.O. Box 11000, FI-00076 Aalto, Finland; jouko.lahtinen@aalto.fi; 3 Department of Physics, University of Jyväskylä, P.O. Box 35, FI-40014 Jyväskylä, Finland; jaakko.julin@jyu.fi

**Keywords:** CaS:Eu, phosphor, photoluminescence, atomic layer deposition

## Abstract

Atomic layer deposition (ALD) technology has unlocked new ways of manipulating the growth of inorganic materials. The fine control at the atomic level allowed by ALD technology creates the perfect conditions for the inclusion of new cationic or anionic elements of the already-known materials. Consequently, novel material characteristics may arise with new functions for applications. This is especially relevant for inorganic luminescent materials where slight changes in the vicinity of the luminescent centers may originate new emission properties. Here, we studied the luminescent properties of CaS:Eu by introducing europium with oxygen ions by ALD, resulting in a novel CaS:EuO thin film. We study structural and photoluminescent properties of two different ALD deposited Eu doped CaS thin films: Eu(thd)_3_ which reacted with H_2_S forming CaS:Eu phosphor, or with O_3_ originating a CaS:EuO phosphor. It was found that the emission wavelength of CaS:EuO was 625.8 nm whereas CaS:Eu was 647 nm. Thus, the inclusion of O^2−^ ions by ALD in a CaS:Eu phosphor results in the blue-shift of 21.2 nm. Our results show that ALD can be an effective way to introduce additional elements (e.g., anionic elements) to engineer the physical properties (e.g., inorganic phosphor emissions) for photonics and optoelectronics.

## 1. Introduction

The development of new luminescent materials and optoelectronic devices such as lamps, displays, lasers, solar cells and photodetectors, relies strongly on the flexibility of the used fabrication technique. The need for higher material quality and versatility in the fabrication of optoelectronic devices was one of the main motivations that lead to the birth and development of atomic layer deposition (ALD) [1,2]. As an example, in the early days of ALD, inorganic phosphors such as ZnS:M (M = Mn, Tb, Tm), SrS:M (M = Ce, Cu, Tb, Pb), CaS:M (M = Eu, Ce, Tb, Pb), were extensively researched for the development of thin film electroluminescent displays (TFEL) [3].

ALD takes a relevant role in the synthetization of inorganic phosphors. The self-limiting mechanism of ALD allows the growth of one atomic layer at the time, consequently leading to accurate control of the thin film thickness and the ability to create multilayer structures [4]. Furthermore, the meticulous inclusion of dopants can be conducted in a single fabrication run, while the host material is being grown. These effects are particularly interesting regarding the doping level control of thin film materials. Thus, they are appropriate for the fabrication of inorganic phosphors, where the doping element acts as a luminescent center. It is known that if the luminescent centers are close to each other it can lead to concentration quenching. However, using ALD, the spatial distance between the luminescent centers can be controlled. The distance is restrained by the steric hindrance effect of the ligands [5,6,7,8]. With this distribution engineering procedure, higher doping concentrations can be achieved in comparison to other fabrication techniques [7], which may reflect in higher luminance values.

In recent years, ALD technology has been used to explore new ways of combining atomic elements to develop materials with unorthodox characteristics. As a result, the research of single-anion (homo-anionic) compounds, such as pure metal oxides, pnictides (nitrides, arsenides, phosphides) or chalcogenides (sulfides, selenides, tellurides), has given place to a mixed anion-centered (multi-anion or hetero-anionic) compounds research [9]. Mixed anion centered materials include for example carbonitrides [10], oxyfluorides [11], oxynitrides [12], oxycarbides [13], and oxysulfides [14]. The ability to incorporate different types of anions allows to change a variety of material properties and characteristics. Electron configuration, electronegativity, size, charge and polarization of this anionic species can lead to the creation of unique structural, chemical, electronic, optical, and magnetic states. In such materials, ALD technique grants the fine-tuning between the combination of cations and multiple anions in thin films [9].

A good example of ALD multi-anion research applied to luminescent materials is a previous study on the control europium of the oxidation state in a Y_2_O_3−x_S_X_ matrix [14]. In this research, it was demonstrated that the oxidation state of Eu luminescent center could be changed by the introduction of Eu atoms with an ALD sub cycle of two different anion precursors: either H_2_S or O_3_. The distinct oxidation states of the luminescent center led to different emission spectrums of the Y_2_O_3−x_S_X_:Eu phosphor.

Inspired by the previous report, we continue to explore the ALD fabrication potential. More specifically, the ability of shifting the peak emission of calcium sulfide doped with europium (CaS:Eu), a well-known red emitting inorganic phosphor material. CaS:Eu luminescent characteristics have been widely studied and used in a variety of applications such as, medical imaging [15,16], cell labeling [17], white-light-emitting diodes [18,19] and electroluminescent devices [20,21,22,23]. The red emission in CaS:Eu is originated from the Eu divalent state of ionization (Eu^2+^) and its broad emission is caused by electron transition from the lower 4f^6^5d^1^ state to the 4f^7^ ground state [24]. This transition in a CaS host originates an emission with wavelength range from 600 to 750 nm and with a peak emission at 650 nm.

In the present article, it is shown that the peak emission of CaS:Eu can be shifted using ALD method. The shift can occur by introducing localized oxygen atoms in the phosphor matrix. Hence, the inorganic phosphor emission was compared between two different structures, (1) pure CaS:Eu, where Eu is exposed to H_2_S reducing gas, and (2) CaS:EuO, where Eu in exposed to O_3_ oxidating gas during the material growth. In comparison to the CaS:Eu phosphor, CaS:EuO reveals a red peak emission towards shorter wavelengths. Similar tuning effects of the CaS:Eu emission has been previously reported by adding alkaline earth metals such as strontium [25,26], magnesium and barium to the reaction mixture [27]. However, we present a study where the tuning is performed by adding oxygen anions reinforcing the idea that ALD can be used to modify the vicinity of the dopant elements. These materials fabricated by ALD can thus be used for applications where the fine color tuning is advantageous such as: red electroluminescent devices [20,21,22,23,28], white-light-emitting diodes [18,19] solar cells [29], and lasers [6,8,30].

## 2. Materials and Methods

**Figure 1:**
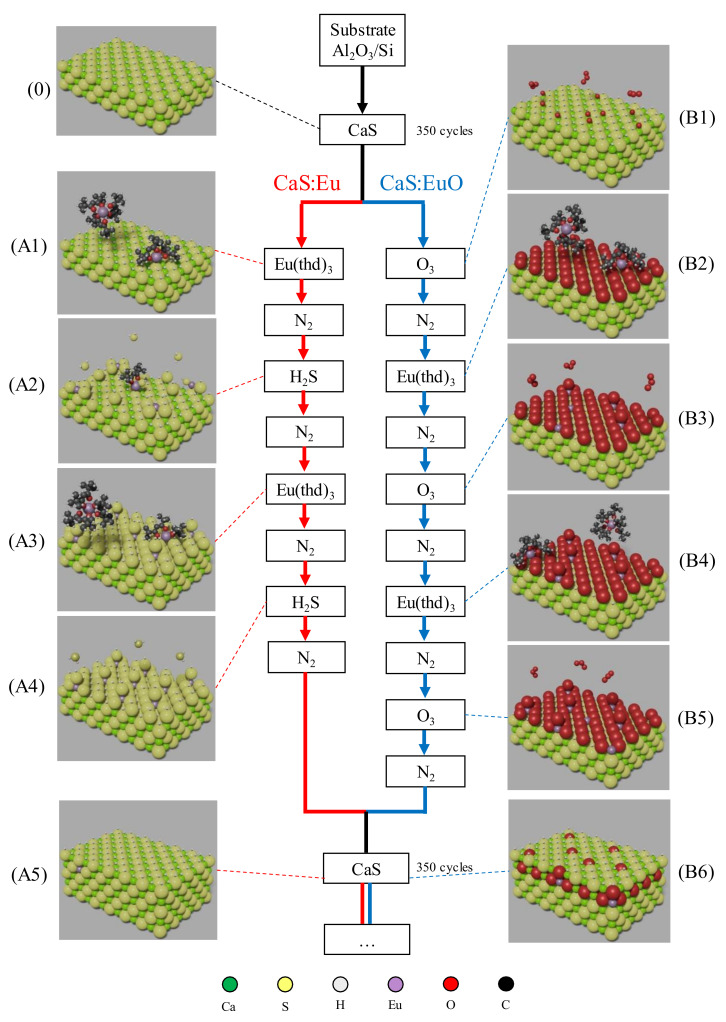
Schematic of ALD doping sequence used in CaS:Eu and CaS:EuO samples. (**0**) Top layers of CaS matrix. Red track represents the doping sequence in CaS:Eu sample including: (**A1**) Eu(thd)_3_ pulsing on CaS layer, (**A2**) H_2_S pulsing after nitrogen purge, (**A3**) second Eu(thd)_3_ pulsing after purge, (**A4**) H_2_S pulsing after nitrogen purge and (**A5**) CaS monolayer after the doping sequence. Blue track represents the doping sequence in CaS:EuO sample including: (**B1**) O_3_ pulse on CaS layer, (**B2**) Eu(thd)_3_ pulsing after purge, (**B3**) second O_3_ pulsing after nitrogen purge, (**B4**) second Eu(thd)_3_ pulsing after purge, (**B5**) third O_3_ pulsing after nitrogen purge and (**B6**) CaS monolayer after the doping sequence. Atomic elements are represented with the following colors: (Ca) green, (S) yellow, (H) gray, (Eu) lavender, (O) red and (C) black.

Film growth was performed using atomic layer deposition in a Beneq TFS-200 ALD-reactor (Beneq Oy, Espoo, Finland). The reactor pressure was around 2 mbar during the process and the temperature was kept at 300 °C. N_2_ was used as a carrier and purge gas. All films were grown on (100)-Si substrates coated with 10 nm of Al_2_O_3_ thin film. Trimethylaluminum (TMA, Al(CH_3_)_3_) (98%, Strem Chemicals UK Ltd., Cambridge, UK) and H_2_O were used to grow Al_2_O_3_ coating film. All CaS based thin films were grown using β-diketonate precursors: Ca(thd)_2_ (99%. Volatec, Porvoo, Finland) and Eu(thd)_3_ (99.5%, Intatrade, Anhalt-Bitterfeld Germany) (THD = 2,2,6,6-tetramethyl-3,5-heptanedionate). Ca(thd)_2_ was kept at 205 °C while Eu(thd)_3_ was kept at 185 °C with a vapor pressure of 133 Pa [31]. Ca(thd)_2_ vapor pressure is unknown in literature. CaS matrix was formed by reacting Ca(thd)_2_ with H_2_S using 350 pulses of each chemical. After the first 350 pulses, Eu(thd)_3_ was introduced as a doping agent by pulsing either H_2_S or O_3_. Two cycles of Eu(thd)_3_ followed by H_2_S were used in one of the samples. A sequence of three O_3_ pulses intercalated by two pulses of Eu(thd)_3_ were used in the other sample. A schematic representation of the doping sequences used in the two samples can be seen in Figure 1. Both phosphor layers were grown using 6 layers of CaS intercalated by five Eu layers, resulting in approximately 100 nm thin film. Finally, another 10 nm layer of Al_2_O_3_ was used to coat the europium doped CaS thin films. Thus, two different samples were studied in this work: Al_2_O_3_/CaS:Eu/Al_2_O_3_/Si and Al_2_O_3_/CaS:EuO/Al_2_O_3_/Si, denominated from now on as CaS:Eu and CaS:EuO, respectively. Table 1 summarizes the pulsing sequence, pulse and purge times, and the respective number of cycles.

The growth per cycle (GPC) of Al_2_O_3_, CaS, Eu_2_O_3_ was determined individually by using a SE400adv ellipsometer (SENTECH Instruments GmbH, Berlin, Germany) with a 633 nm wavelength at 70° angle of incidence. The crystallinity of europium doped CaS thin films was investigated by X-ray diffraction (XRD) using the Cu Kα line in a Rigaku SmartLab (Rigaku Europe SE, Neu-Isenburg, Germany) high-resolution X-ray diffractometer. Data acquired by the XRD were analyzed using the HighScore Plus 4.6 (PANalytical B.V., Almelo, The Netherlands). The X-ray photoelectron spectroscopy (XPS) measurements were made using Kratos Axis Ultra system (Kratos Analytical Ltd., Manchester, UK), equipped with a monochromatic Al Kα X-ray source. All measurements were performed with 0.3 mm × 0.7 mm analysis area. The wide scans were performed with 80 eV pass energy and 1 eV energy step, and the high-resolution scans were performed with 40 eV pass energy at 0.1 eV steps. Eu doped CaS samples were analyzed with elastic recoil detection analysis (ERDA) using a 13.6 MeV ^63^Cu^7+^ ion beam. The investigated area was approximately 2 × 2 mm^2^. The measurement was carried out with a 70° angle between the sample normal and beam, whereas the scattering angle was 40.6°. Time-of-flight–energy (ToF-E) telescope was used to detect the recoiling atoms from the sample. Photoluminescence (PL) emission of Eu doped CaS thin films was measured with a Hitachi F-7100 Fluorescence Spectrophotometer (Hitachi High-Tech Analytical Science Ltd., Abingdon, UK) equipped with a 150 W xenon lamp. Measurements were performed at room temperature with a photomultiplier tube voltage of 400 V, with a bandwidth of 10 nm.

## 3. Results

### 3.1. Crystallinity

**Figure 2:**
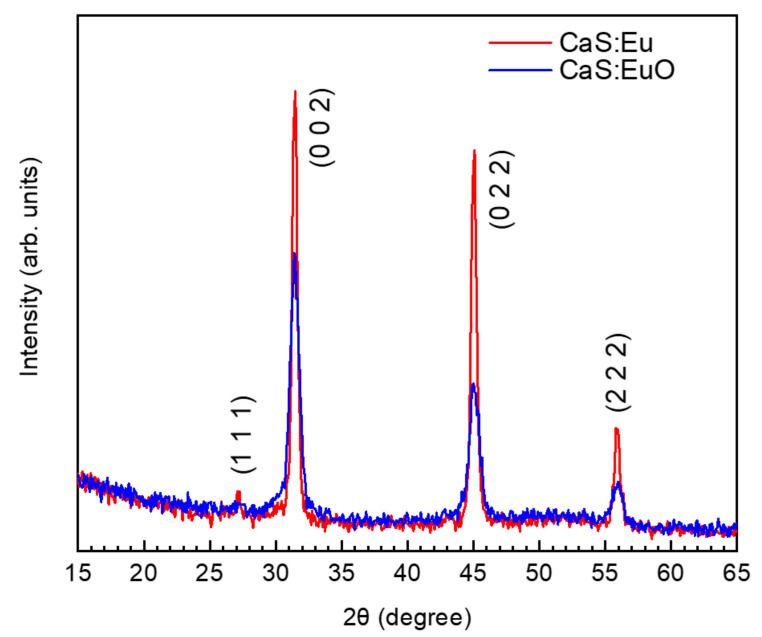
Grazing incidence XRD of CaS:Eu and CaS:EuO samples.

Figure 2 illustrates grazing incidence XRD patterns for CaS:Eu and CaS:EuO thin films measured between 15° and 65°. XRD patterns were carried out with an incidence angle of 2°. The grazing incident XRD data for both thin films show a main polycrystalline phase (randomly orientated). The identified peaks are related to (1 1 1), (0 0 2), (0 2 2) and (2 2 2) reflections. The peak position and the order of peak intensity are similar to the one reported by cross-reference COD:96-900-8607 [32]. Thus, indicating that CaS:Eu and CaS:EuO have a cubic structure with Fm$\bar{3}$m space group. The lattice parameter was determined to be a = 5.68 Å and a = 5.69 Å for CaS:Eu and CaS:EuO, respectively. Furthermore, CaS:Eu shows a higher level of crystallinity compared to CaS:EuO thin film.

### 3.2. Chemical Analysis

XPS was performed on both CaS:Eu and CaS:EuO samples. We used argon ion etching to remove the 10 nm Al_2_O_3_ top coating layer prior to detailed analysis of the materials. The removal of the oxide was followed by XPS measurements after 100 s, 200 s, 400 s, 600 s and 1000 s of ion etching. In the CaS:Eu sample the Al 2p peak disappeared after 600 s whereas in the CaS:EuO sample the Al 2p intensity decreased by a factor of four after 1000 s.

**Figure 3:**
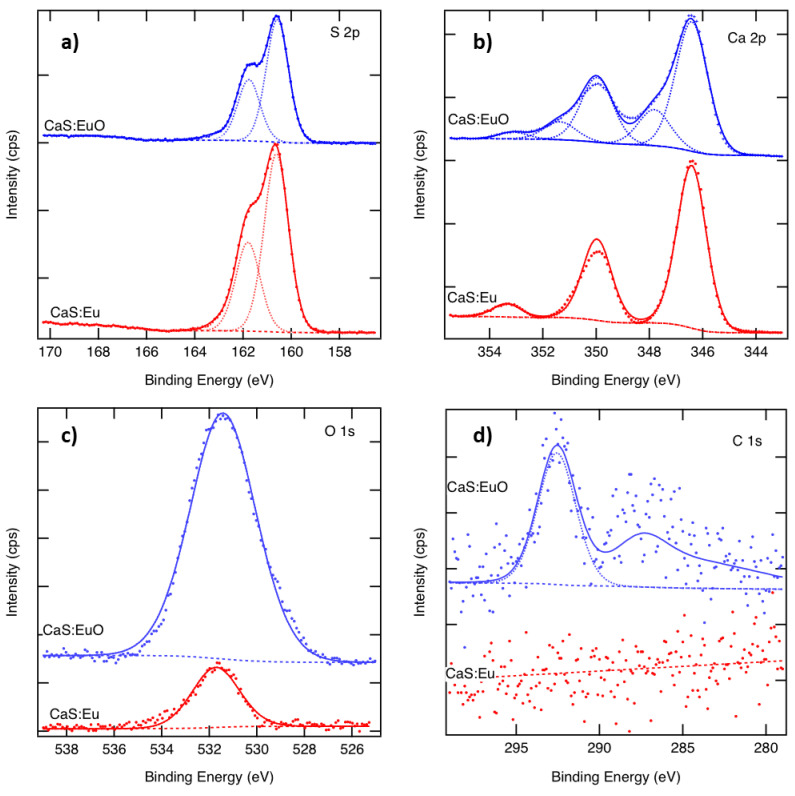
Measured and fitted (**a**) S 2p, (**b**) Ca 2p and (**c**) O 1s and (**d**) C 1s spectra of CaS:Eu and CaS:EuO samples.

The S 2p spectra of CaS:Eu and CaS:EuO in Figure 3a, show a typical doublet with 1.18 eV separation. The S 2p binding energy of 160.7 eV indicates the presence of sulfides, whereas the sulfate component around 169 eV is missing [33]. The Ca 2p spectra of both samples are illustrated in Figure 3b. The CaS:Eu spectrum shows a typical doublet with 3.55 eV separation. The Ca 2p_3/2_ binding energy of 346.4 eV corresponds to CaS or CaO. In the CaS:EuO sample, an additional component related to the CaCO_3_ or CaSO_4_ is seen at 347.9 eV, with a peak area corresponding to 20% of the total Ca. As there is no sulphate peak in the S 2p spectra, we call this peak CaCO_3_ although the binding energy would better fit to 348.0 eV of CaSO_4_ than (347.1 ± 0.3) eV of CaCO_3_ [33]. The fitting also includes a satellite peak above 353 eV in both samples. Figure 3c shows the measured O 1s spectra. There is only one peak appearing at 531.6 eV in both samples, indicating similar oxygen. However, the O 1s signal from CaS:EuO indicates 42 at-% of oxygen whereas that of the CaS:Eu only 10 at-%. Figure 3d shows the C 1s spectra. The CaS:Eu sample does not contain carbon. In the CaS:EuO sample a small C 1s peak around 292 eV is seen. This peak can be related to CO_3_ groups. There might also be some structure above the noise level around 286 eV. The amount of carbon in the CaS:EuO sample is below 2 at-%.

**Figure 4:**
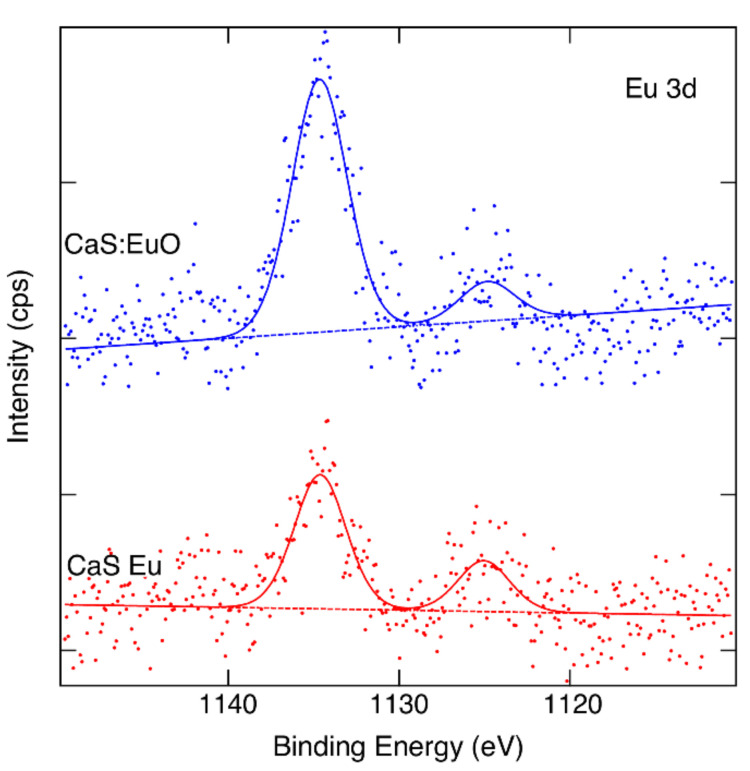
Measured and fitted Eu 3d spectra of CaS:Eu and CaS:EuO samples.

Figure 4 illustrate the Eu 3d core levels of CaS:Eu and CaS:EuO, respectively. Eu^3+^ and Eu^2+^ oxidation states can be identified in both samples if we accept binding energies 1134.4 eV and 1124.7 eV for the components [34]. However, the data are noisy despite of the long measurement time used. We can estimate that the Eu^3+^/Eu^2+^ ratio is approximately 5/2 in CaS:Eu whereas in CaS:EuO it is close to 6/1.

**Figure 5:**
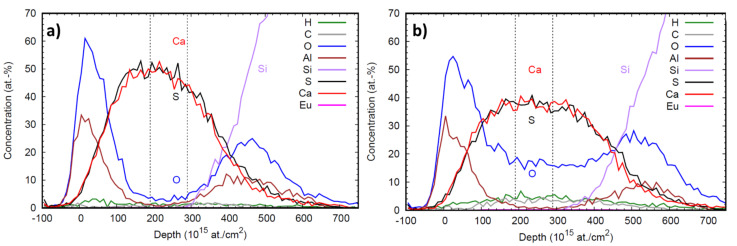
Depth profile of (**a**) CaS:Eu and (**b**) CaS:EuO samples. Dashed vertical lines indicate the depth range used for elemental compositional analysis.

Figure 5a,b illustrates the elemental depth profile obtained by ToF-ERDA measurements of CaS:Eu and CaS:EuO samples, respectively. The depth resolution degrades deeper in the sample due to energy loss straggling. Therefore, the elemental composition of both europium doped CaS layers was analyzed by selecting an interval representative of the film bulk, with no Al and high concentration of Ca and S. The vertical lines in the depth profile plots indicate the depth range from which the averages in Table 2 are calculated.

Table 2 shows a comparison between the element composition of CaS:Eu and CaS:EuO layers. In both films, the atomic percentages of S and Ca are approximately the same, demonstrating that CaS is present with a stoichiometry of 1:1. The doping level is higher for samples where Eu is grown on CaS with ozone. As expected, the level of oxygen present in the doped layer is higher when ozone is used to introduce Eu(thd)_3_. However, in the sample where no ozone was used there is still a low concentration of oxygen impurities. Both H and C contamination levels were higher in the CaS:EuO sample.

### 3.3. Photoluminescence

**Figure 6:**
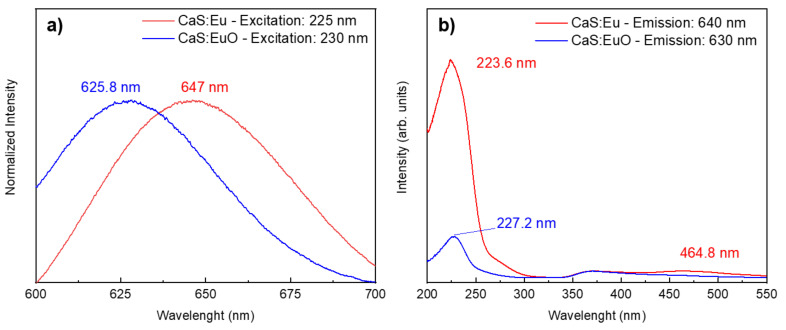
(**a**) Normalized emission spectra and (**b**) excitation spectra from CaS:Eu and CaS:EuO, measured at room temperature.

The normalized PL spectra were plotted in Figure 6a to facilitate the comparison between the maximum emission wavelength of CaS:Eu and CaS:EuO samples. Both photoluminescent emission spectra were measured between 600 and 700 nm and excited with approximated wavelengths values correspondent to the maximum emission intensity. The CaS:Eu sample has a maximum emission value of 647 nm, when excited with a wavelength of 225 nm. The maximum emission is related to the Eu^2+^ ion Laporte allowed transition, from 4f^6^ 5d^1^ to the 4f^7^ (^8^S_7/2_), characteristic of CaS:Eu^2+^ materials [24]. In addition, CaS:EuO sample shows a maximum peak emission of 625.8 nm, for an excitation wavelength of 230 nm. The PL measurements were carried out in three different CaS:EuO samples for reproducibility proposes. All tree samples showed similar behavior.

Furthermore, photoluminescent excitation (PLE) spectra in Figure 6b were registered by monitoring the emission at 640 nm and 630 nm for CaS:Eu and CaS:EuO samples, respectively. All samples show a broad excitation band between 200 and 300 nm, which can be associated with the Eu^2+^ 4f^7^ (^8^S_7/2_) → 4f^6^ 5d^1^ [E_g_] and valence-to-conduction band transitions. Whereas the low intensity excitation band in CaS:Eu sample from 400 to 550 nm is related to Eu^2+^ 4f^7^ → 4f^6^ 5d^1^ [t_2g_] transition [35]. This transition is believed to have too low intensity to be detected in CaS:EuO sample, therefore it was not used to excite the samples.

A summary of Figure 6a,b is presented in Table 3, which include the excitation and absorption wavelength; excitation and emission peak; and respective intensities. In Table 3, it is clear that CaS:Eu sample has higher intensities in comparison to CaS:EuO sample, regarding excitation and emission peaks.

## 4. Discussion

With the data acquired from XPS it is possible to assume that the Al from the top coating layer can be measured until 600 s of argon sputtering, in CaS:Eu sample. Thus, leading to the conclusion that beyond the 600 s etching step, a nearly pure CaS:Eu layer can be found. Faint traces of oxygen can still be analyzed within the europium doped CaS layer. These traces of oxygen could originate from the O diffusion of the Al_2_O_3_ top coating layer into the CaS:Eu layer. In this case, the oxygen contamination can occur due to the slow ALD fabrication process at 300 °C. Additionally, due to the high Ca affinity towards oxygen atoms, there is the possibility of O present in the β-diketonate molecule to be adsorbed on the CaS surface, upon Eu(thd)_3_ and H_2_S reaction. The same oxygen contamination can be visualized in the ToF-ERDA analysis, see Table 2. As expected, the presence of oxygen in CaS:EuO sample can be identified clearly after the 600 s due to the intentional introduction of O atoms. XPS analysis of sulfur species revealed that sulfur is detected in form of sulfide and no sulfates are present in the phosphor layer.

Furthermore, the results from XPS and ToF-ERDA show higher level of carbon content within the CaS:EuO sample. These data are supported by the detection of CaCO_3_ component in Figure 3b and higher carbon concentration shown in Table 2. The carbon contamination in CaS:EuO could be explained by the reaction between ozone and the β-diketonate precursor (Eu(thd)_3_), which is known to originate carbonates in the films [14]. Additionally, the ToF-ERDA analysis revealed a lower hydrogen presence in CaS:Eu, which indicates the excellent reactivity between Ca(thd)_2_ and Eu(thd)_3_ with H_2_S. These efficient surface reactions are responsible to effectively remove lighter species, such as H [36]. In contrast, higher levels of carbon and hydrogen contamination in CaS:EuO sample can be correlated with the lower reactivity of O_3_ species with Ca(thd)_2_ and Eu(thd)_3_, in comparison to H_2_S. Moreover, the intentional inclusion of oxygen atoms into the europium doped CaS layer may be the major responsible factor for the lower level of crystallinity. The presence of O^2−^ can cause distortions in the CaS crystal lattice leading to structural defects. Nonetheless, the crystal structure of CaS:EuO is still similar to CaS:Eu and no traces of CaO or CaSO_x_ polycrystalline phases are identified.

It was found that the oxidation level ratio of Eu^3+^/Eu^2+^ is higher in the CaS:EuO samples. The higher Eu^3+^ ion presence correlates with the presence of O^2−^ ions, which is expected since Eu tends to oxidize to its trivalent form whenever enough oxygen atoms are available. Furthermore, the europium doping level was revealed to be slightly higher when Eu(thd)^3^ was grown on the CaS layer with ozone instead of H_2_S. This difference in the doping level may be explained by the facilitated introduction of Eu atoms when there is a monolayer of oxygen present in the CaS surface. Oxygen has higher electronegativity than sulfur and thus increases the probability of Eu adsorption. Note that, Eu(thd)_3_ is pulsed into the ALD reactor chamber after an O_3_ pulse. In a previous study it was stated that Eu may be more reactive towards the H_2_S in comparison to ozone, and thus increasing the doping level [14]. However, in the present study, the surface has an oxygen monolayer instead of a sulfur monolayer, prior to the Eu(thd)_3_ pulse. The oxygen presence in the surface may be a determinant factor for the Eu growth rate as a dopant, as it may increase the adsorption of Eu(thd)_3_.

**Figure 7:**
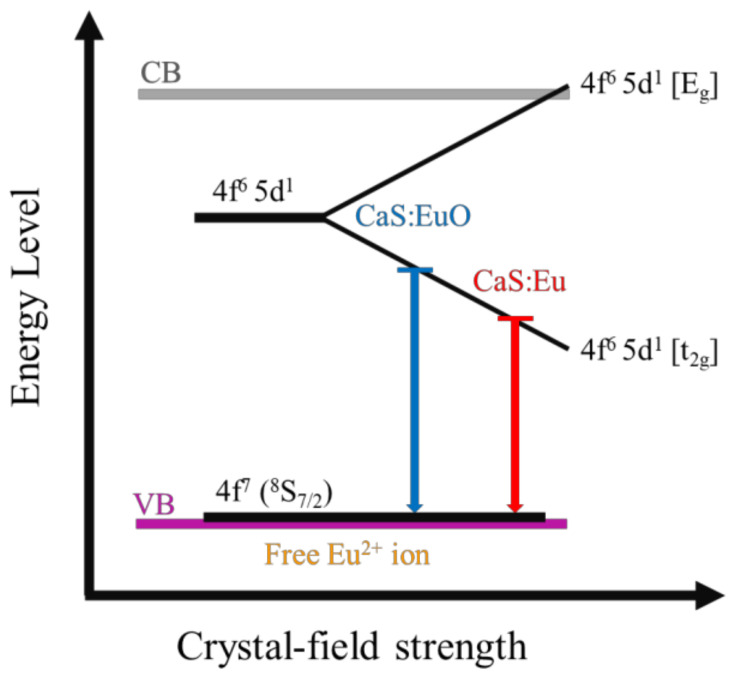
Schematic energy diagram of Eu²⁺ 5d level splitting as function of crystal-field strength in CaS:Eu and CaS:EuO samples.

The different emission wavelengths of CaS:Eu and CaS:EuO samples can be explained by the presence of O^2−^ ion in the phosphor layer. It is probable that O^2−^ ion inclusion originated structural defects in the CaS structure. This can be concluded due to the lower intensity of the PL emission in the CaS:EuO sample, since certain defects may decrease luminance. Additionally, the defects may have distorted the crystal structure which led to a larger lattice constant, assuming a value of 5.69 Å instead of 5.68 Å in the presence of O^2−^ ions. The larger lattice constant may indicate that the distance between Eu^2+^ cations and the anions increase. The larger distance between Eu^2+^ cations and the anions lead to a decrease in the crystal-field strength [25,26]. Therefore, the addition of a O^2−^ ion originates a decrease in the energy difference between t_2g_ and the E_g_ states of the 4f^6^ 5d^1^ electronic configuration, thus leading to blue-shifted emission. Figure 7 illustrates a schematic of Eu^2+^ energy level as function of the crystal-field. Several factors may explain the lower emission intensity of the CaS:EuO sample in comparison to CaS:Eu, such as slightly lower Eu concentration and lower crystallinity.

## 5. Conclusions

In this work, two samples of Eu doped CaS phosphors were grown by ALD. Structural, chemical, compositional and emission properties of both samples were correlated with the fabrication conditions. XRD analysis shows the polycrystalline nature of the phosphor layers with a slight difference in the lattice dimension and level of crystallinity between the samples. XPS was used to detect the chemical bounds between the ionic species present in the phosphor layer. Additionally, ToF-ERDA accurately identify the levels of contamination originated during the ALD fabrication process. It was shown that the inclusion of Eu together with oxygen as a dopant in the CaS matrix reduces the overall crystal-field strength, which leads to blue-shifted emission by 21.2 nm in comparison to the CaS:Eu sample. The peak emission is found at 625.8 nm for CaS:EuO, whereas CaS:Eu shows the typical emission around 647 nm. These results show that by introducing oxygen anion in the CaS:Eu thin film using ALD method, the resulting phosphor emission can be modified.

## Figures and Tables

**Table 1:** Pulsing sequences and respective pulsing times, number of cycles per layer for CaS:Eu and CaS:EuO phosphor layers.

Sample	Pulsing Sequence	Pulsing Time (s)	Number of Cycles per Layer
CaS:Eu	Ca(Thd)_2_/N_2_/H_2_S/N_2_	2/5/0.2/3	350
	Eu(Thd)_3_/N_2_/H_2_S/N_2_	3/7/0.2/3	2
CaS:EuO	Ca(Thd)_2_/N_2_/H_2_S/N_2_	2/5/0.2/3	350
	O_3_/N_2_/Eu(Thd)_3_/N_2_/O_3_/N_2_/ Eu(Thd)_3_/N_2_/O_3_/N_2_	3/5/3/7/3/5/3/7/3/5	1

**Table 2:** Elemental composition of the CaS:Eu and CaS:EuO samples measured with ToF-ERDA.

Sample	H (at. %)	C (at. %)	O (at. %)	S (at. %)	Ca (at. %)	Eu (at. %)
CaS:Eu	1.2 ± 0.2	0.8 ± 0.3	3.2 ± 0.3	47.7 ± 0.5	47.0 ± 0.5	0.13 ± 0.02
CaS:EuO	4.7 ± 0.3	3.4 ± 0.3	16.8 ± 0.5	37.5 ± 0.5	37.4 ± 0.5	0.17 ± 0.02

**Table 3:** Summary of the CaS:Eu and CaS:EuO emission and excitation data acquired from the photoluminescent measurements.

Sample	Excitation Wavelength [nm]	Peak Emission [nm]	Intensity (arb. un.)	Emission Wavelength [nm]	Peak Excitation [nm]	Intensity (arb. un.)
CaS:Eu	225	647	93.61	640	223.6	89.17
CaS:EuO	230	625.8	19.87	630	227.2	20.49

## Data Availability

Data sharing is not applicable to this article.
